# Supplementary material for: Microbiota composition and mucosal immunity in patients with asymptomatic diverticulosis and controls
Source: PLoS One. 2021 Sep 7;16(9):e0256657. doi: 10.1371/journal.pone.0256657 (PMC8423250; doi:10.1371/journal.pone.0256657)
Supplement: Attachment — Submitted filename: Response to Reviewers 20210702.docx [file pone.0256657.s003.docx]

Amsterdam, 02-07-2021

Dear Mr Staley,

Thank you for the review of our manuscript entitled “Microbiota composition and mucosal inflammation in patients with asymptomatic diverticulosis and controls” (PONE-D-21-08956). With these valuable comments we were able to improve our manuscript.

Below I copied the reviewers’ comments and our response to each comment.

We look forward to your response and hope that you find our revised manuscript eligible for publication in *PLOS ONE*.

Sincerely,

Tess van Rossen

PhD candidate, department of Medical Microbiology & Infection Control, Amsterdam UMC

---------------------------------------------------------------------------------------------------------------------------------

**Reviewer comments:**

**Reviewer #1:**

**Excellent manuscript showing that the presence of asymptomatic diverticulosis is not associated with significant alterations of intestinal mucosal microbiota. The findings suggest that gut microbiota should not have a major role in the pathogenesis of diverticula. The topic is not completely novel, since there are already some studies investigating the same research question (correctly acknowledged in the discussion). However, the present study has been conducted on a larger sample size and with a sounder methodology than its counterparts.**

**I have just three comments/suggestions:**

1) **Cases (patients with diverticulosis) and controls were not matched by age. This is an important limitation of the study. In fact, age is generally considered one of the main determinants of intestinal microbiota composition. However, the effect of age on microbiota begins to be relevant after the age of 70-75 years old, especially if no significant comorbidities or disability exist. Thus, the effect of different age on the microbiota composition of the two groups enrolled in this study could have been negligible, provided that the burden of comorbidities and disability of the two groups was similar. I suggest that the authors include a comment on these issues in their manuscript.**

Thank you for the review of our manuscript and for your positive response. We agree that this is a limitation of the study. As you suggested, we included a comment and corresponding references in the discussion section (lines 327-333):

“Our study is hampered by several drawbacks. Firstly, our sample size is small and AD patients and controls were not matched by age. As AD is commonly found with increasing age, our study is not powered to find subtle, age-dependent differences in microbiota composition. Age is generally considered one of the main determinants of intestinal microbiota composition; however this begins to be relevant after the age of 70-75 years old, especially if no significant comorbidities or disability exist (21,22). This is in line with our finding that although the AD group was significantly older than the control group (66 vs 56 years respectively), the microbiota composition did not differ between the two groups.”

In addition, we re-assessed electronic patient records of included patients to evaluate potential differences in comorbidities and disability of the two groups. We found that the burden of disability was similar in the two groups. The results are displayed in Table 1 and included in the Results section (lines 175-179):

“The number of patients with specific comorbidities was similar in the two groups, except that gastro-intestinal disorders and malignancies (but not colon cancer) were more frequent in the control group compared to the AD group. Gastro-intestinal comorbidity in the control group concerned esophagitis (n=2), aspecific colitis (n=1) and irritable bowel syndrome (n=1).”

2) **No prespecified sample size calculation seems to have been performed for this study. Please specify that the study was conducted on a convenience sample size.**

Thank you for this suggestion, we included this statement in the Methods section (lines 87-88).

3) **It would have been interesting to include in the study also an analysis of the fecal microbiota of participants, to verify whether the composition of the mucosal and fecal microbiota was consistent. Fecal microbiota is much more accessible for analyses, also from a clinical perspective.**

We agree that an additional analysis of fecal samples (or rectal swabs, also a practical sample type from a clinical perspective) would have been very interesting for comparison between the luminal and fecal microbiota composition. Unfortunately, feces was not collected in this study but we included this suggestion/limitation in the Discussion section (lines 352-356):

“Another option might be to use rectal swabs or feces to verify whether the composition of the mucosal and fecal microbiota was consistent. Furthermore, collection of rectal swabs or feces is more practical and patient-friendly, although these methods have their own disadvantages as described before (24, 25).”

**Reviewer #2:**

**The topic of this manuscript is very interesting. The pathophysiology of diverticular disease is still incompletely known and the role of microbiota and immunity is only partially reported in the literature. This study evaluated mucosal microbiota and immunity in asymptomatic diverticulosis compared with a control group without diverticula. There are, however, several limitations that make it difficult to support the conclusions speculated by the authors and make the results unreliable. Among the exclusion criteria, the authors did not consider the use of antibiotics and laxatives which are factors changing gut microbiota. The lack of information about diet generates doubts about the results.**

Thank you for the review of our manuscript your valuable suggestions.

Concerning the exclusion criteria, patients with antibiotics and laxative use were indeed not excluded beforehand. However, we re-assessed electronic patient records of our study population and found that none of the patients received antibiotics, and only few patients used laxatives (AD group: 3 patients, control group: 1 patient). This data is added to the text of the Results section and Table 1 (page 9). Furthermore, all patients underwent routine bowel lavage (high dose laxatives) for colonoscopy so we assume that the potential effect of prior laxative use on the mucosal microbiota composition is probably marginal compared to the effect of the bowel preparation. Unfortunately, data on diet was lacking, since this factor is not regularly reported in patient records. We addressed this limitation in the Discussion section (lines 334-336):

“We only had limited availability to patient demographics. Several other factors such as diet can influence microbiota composition for which should be corrected (23)."

**Asymptomatic diverticulosis and controls significantly differ for age, which again influences gut microbiota. Without correcting data for this variable, there is likely a bias in the results.**

Thank you for this comment. See also the answer on comment 1 of reviewer #1: We agree that this is a limitation of the study. However, since the microbiota composition of the AD group and control group was comparable despite the difference in age, we do not expect that age has influenced the results. Furthermore, previous studies show that the effect of age on the microbiota composition begins to be relevant after the age of 70-75 years old, while the age of the AD patients and controls was 66 and 56 years old, respectively. Nevertheless, we included this limitation and corresponding references in the discussion section (lines 327-333):

“Our study is hampered by several drawbacks. Firstly, our sample size is small and AD patients and controls were not matched by age. As AD is commonly found with increasing age, our study is not powered to find subtle, age-dependent differences in microbiota composition. Age is generally considered one of the main determinants of intestinal microbiota composition; however this begins to be relevant after the age of 70-75 years old, especially if no significant comorbidities or disability exist (21,22). This is in line with our finding that although the AD group was significantly older than the control group (66 vs 56 years respectively), the microbiota composition did not differ between the two groups.”

**Concerning immunity, the study did not look at macrophages, which as authors themselves reported in the manuscript, have been found to be different in diverticular disease compared to controls.**

Thank you for this comment. We agree that analysis of macrophages would have been an interesting addition to our study. Unfortunately, this data was not collected in our study set, but we addressed this limitation and suggestion for future studies in the Discussion section (lines 356-358):

“An assessment of other immune cells such as macrophages, or cytokines in our data set could provide a more detailed view of the mucosal immunity in patients with AD and should be evaluated in future studies.”

**Additional Editor Comments:**

**The study addresses an important and emerging question concerning the role of the intestinal microbiota in diverticular disease. As both reviewers acknowledge, age is an important confounding factor, in addition to other exclusion requirements ie, antibiotic exposure or other medications and clinical metadata including immunological assays or dietary surveys are needed to properly contextualize these data. Without these, this is a well conducted survey but provides little novel information.**

Thank you for the review of our manuscript and the positive feedback. We agree that the metadata is important for correct interpretation of the results on the role of the intestinal microbiota in diverticulosis and diverticulitis. Therefore, we re-assessed electronic patient records to extract data on potential confounders, such as smoking, alcohol use, antibiotic exposure, and comorbidities. We found no large differences between the two groups. The results are displayed in Table 1 and included in the Results section (lines 173-179):

“Six out of 19 AD patients were smokers, compared to 3/24 controls, and alcohol consumption was slightly higher in the AD group compared to the control group. None of the patients received antibiotics via the hospital prior to colonoscopy. The number of patients with specific comorbidities was similar in the two groups, except that gastro-intestinal disorders and malignancies (but not colon cancer) were more frequent in the control group compared to the AD group. Gastro-intestinal comorbidity in the control group concerned esophagitis (n=2), aspecific colitis (n=1) and irritable bowel syndrome (n=1).”
